# Heavy Metal Complexation of Thiol-Containing Peptides from Soy Glycinin Hydrolysates

**DOI:** 10.3390/ijms16048040

**Published:** 2015-04-10

**Authors:** Xiuzhen Ding, Yufei Hua, Yeming Chen, Caimeng Zhang, Xiangzhen Kong

**Affiliations:** State Key Laboratory of Food Science and Technology, School of Food Science and Technology, Jiangnan University, Wuxi 214122, China; E-Mails: ding_xiu_zhen@126.com (X.D.); yfhua@jiangnan.edu.cn (Y.H.); chenyeming19821213@gmail.com (Y.C.); cmzhang@jiangnan.edu.cn (C.Z.)

**Keywords:** thiol-containing peptides, soy glycinin hydrolysates, complexation, Hg^2+^, Cd^2+^, Pb^2+^

## Abstract

Many thiol-containing molecules show heavy metal complexation ability and are used as antidotes. In this study, the potential function associated with thiol-containing peptides (TCPs) from soy protein hydrolysates as natural detoxicants for heavy metals is reported. TCPs enriched by Thiopropyl-Sepharose 6B covalent chromatography had different molecular weight distributions as well as different numbers of proton dissociable groups, depending on the proteases and degree of hydrolysis. The major contribution of sulfhydryl groups was confirmed by the largest pH decrease between 8.0 and 8.5 of the pH titration curves. The complexation of TCPs with heavy metalswas evaluated by stability constants (β_n_) of TCP-metal complexes whose stoichiometry was found to be 1:1 (ML) and 1:2 (ML_2_). TCPs from degree of hydrolysis of 25% hydrolysates gave high affinities towards Hg^2+^, Cd^2+^, and Pb^2+^ (giving similar or even bigger lgβ values than that of glutathione). A significantly positive correlation was found between the logarithm of stability constants for ML_2_ (lgβ_2_) and the sulfhydryl group content. Molecular weight distribution of TCPs affected the complexation with Pb^2+^ notably more than Hg^2+^ and Cd^2+^. These results suggest that soy TCPs have the potential to be used in the formulation of functional foods to counteract heavy metal accumulation in humans.

## 1. Introduction

With the rapid industrialization and urbanization, pollution from heavy metals has become a serious threat to public health. It has been well established that the nonessential heavy metals such as mercury, cadmium, and lead can be highly toxic even at low concentration leading to the dysfunction of kidneys, liver, heart, and immune and nervous systems through generation of reactive radicals, which result in cellular damage such as depletion of enzyme activities, as well as damage to lipid bilayers and DNA in the human body [1,2]. As such, it is significant to develop effective and safe detoxifying agents in order to prevent metal accumulation in individuals.

Peptides with an abundance of Cys residues are known to bind heavy metals with high affinity [3]. Sulfur-rich metal-sequestering peptides, such as glutathione (GSH), metallothioneins (MTs) and phytochelatins (PCs) are very important to biological defense strategies against heavy metal poisoning [4]. Glutathione is the most abundant non-protein thiol in biological systems with intracellular concentrations of between 0.1 and 10 mmol·kg^−1^ present in microorganisms, fungi, and plant and animal tissues [5,6]. MT is a super-family of cysteine-rich low molecular weight (6–7 kDa) proteins or polypeptides containing some 60 amino acid residues among which is 20 Cys. It binds a total of seven equivalents of bivalent metal ions [7]. PCs are a family of short cysteine-rich metal-chelating peptides with a structure of γGlu-Cys_n_Gly, where, n varies from 2 to 11 depending on the species and conditions of exposure to metal ions [8]. They primarily form a *M*w 3600 Da complex with cadmium [9]. Coordination to cysteine residues is the dominant mechanism for these peptides to sequester heavy metals [10]. Sulfur donor atoms are also found at the metal-binding sites of many metalloenzymes and proteins, and bind tightly to various soft metal ions [11].

Chelation therapy is the preferred medical treatment for reducing the toxic effects of heavy metals. Most of the chelating agents have been compounds containing thiols, such as d-penicillamine, dimercaptosuccinic acid, 2,3-dimercaprol, dimercaptopropane-sulfonic acid, and when necessary cystein (Cys) and *N*-acetylcysteine (NAC) [12]. However, most of the conventional chelators are compromised with many side effects and drawbacks [12]. Maria, N.S. [13] indicated that TCPs were viable alternatives to Cys and NAC. Other synthesized TCPs [14,15,16] also showed effective detoxification against heavy metal toxicity. On the other hand, synthesized compounds have been generally recognized as having potential health risks, while natural ones from food have attracted increasing interest. However, few researches on TCPs from native sources have been reported.

Soy proteins are abundant and a relatively inexpensive source of protein having high nutritional value and excellent functional properties. They have been widely utilized in the food industry as a major ingredient. Morever, soy protein hydrolysates have been extensively applied in food products because of their various bioactivities [17,18,19,20,21,22], such as antihypertensive, hypocholesterolemic, antiobesity, antioxidant, anticancer, and immunomodulatory. Soy glycinin (11S) is one of two major components of soy proteins and contains 41 mol/mol Cys residues [23]. Protease treatment could release thiol-containing peptides (TCPs). It is scientifically and economically valuable to know if TCPs in 11S hydrolysates (SGHs) display heavy metal chelating ability.

Thiopropyl-Sepharose 6B, based on thiol-disulfide exchange, has been successfully applied to the extraction of thiol-containing peptides or proteins in the proteome of mouse brain [24], human mammary epithelial cell [25] and rat myocardial redox [26]. However, there are only limited studies concerning thiol-containing peptides from food protein hydrolysates. Dionysius *et al.* [27] reported the purification and characterization of antibacterial peptides from bovine lactoferrin hydrolysates from which, three thiol-containing peptides with antibacterial activity toward enterotoxigenic *Escherichia coli* were found, using continuous chromatography. A cysteine-rich antimicrobial peptide, CgPep33, exhibiting activity against Gram-positive and Gram-negative bacteria and fungi, was isolated from the alcalase and bromelin hydrolysates of oyster [28].

Stability constants of the heavy metal-ligand complexes are the most often used parameters to evaluate the effectiveness of antidotes for heavy metal poisoning *in vitro*. Qualitatively, the greater the stability constant, the greater is the effectiveness of the antidote. The potentiometric titration method is the commonly used approach to determine the stability constants because of its accuracy and accessibility. Atabey *et al.* [29] and Gamov *et al.* [30] determined the stability constants of aurintricarboxylic acid, and nicotinamide-heavy metal complexes using the potentiometric titration method. Leverrier *et al.* [31] studied the interaction of glutathione with Cd^2+^ by combination of classical potentiometric titration and spectroscopic methods. Cardiano *et al.* [32] used the potentiometric method to study the stability constants between Hg^2+^ and thiols and ^1^H NMR to confirm the results. Ngu-Schwemlein *et al.* [13] evaluated the mercury (II) binding affinities and associated thermodynamic parameters of cysteine, histidine, tryptophan, and their di- and tri-peptides by isothermal titration calorimetry (ITC). The traditional methods for detection of heavy metal ions include atomic absorption/emission spectroscopy, inductively-coupled plasma mass spectrometry, and ion-selective electrodes. Recent trends involve the application of nanomaterials, such as a G-quadruplex-based platform for the detection of Hg^2+^ ions using a luminescent iridium(III) complex which functions effectively in real water samples under conditions of low turbidity and low metal ion [33,34].

Up to now, reports on the bioactivity of TCPs in SGHs have been limited. In the present work, soy glycinin was hydrolyzed by different enzymes to different degrees of hydrolysis and TCPs in SGHs were enriched by covalent chromatography. The complexing ability of TCPs with Pb^2+^, Cd^2+^, and Hg^2+^ was evaluated by stability constants of TCP-metal complexes comparing with glutathione. The effects of sulfhydryl group content and molecular weight (*M*w) distribution on the complexation were also discussed. The results of this study may provide insight into soy protein hydrolysates as detoxication agents for heavy metal poisoning.

## 2. Results and Discussion

### 2.1. Characterization of Soy Glycinin Hydrolysates and Thiol-Containing Peptides (TCPs)

**Figure 1:**
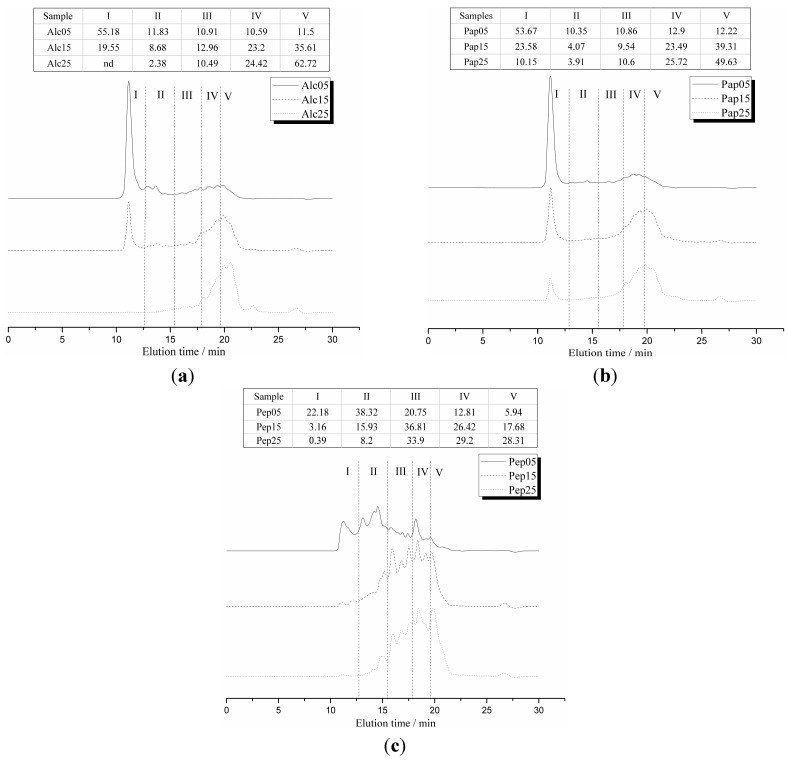
SEC-HPLC profiles of soy glycinin hydrolysates (SGHs) with different degree of hydrolysates (DH) obtained by enzymatic hydrolysis with alcalase (**a**), papain (**b**) and pepsin (**c**). The SEC-HPLC profiles are divided into 5 fractions. Inserted tables give the percentage of every fraction. Fraction I corresponds to protein fragments with molecular weight (*M*w) exceeding 10 kDa, fraction II to protein fragments with *M*w between 3 and 10 kDa, fraction III to protein fragments with *M*w between 1 and 3 kDa, fraction IV to protein fragments with *M*w between 0.5 and 1 kDa and fraction V to material with *M*w lower than 0.5 kDa. Sample codes consist of 3 letters encoding the enzyme and 2 digits encoding the DH.

SGHs with different degrees of hydrolysis were prepared by proteolysis of glycinin with three commercially available proteases. The *M*w distribution of SGHs was determined by SEC-HPLC. The elution curves were divided into five fractions, which corresponded to a *M*w range of >10,000, 10,000–3000, 3000–1000, 1000–500 and <500 Da, respectively (Figure 1). For alcalase and papain, two enzymes, which acted at neutral to slight alkaline pH, their hydrolysates at degree of hydrolysis(DH) of 5% and 15% showed a remarkable peak of *M*w >10,000 Da, which might have been induced by the aggregation of small peptides by hydrophobic interactions. At DH of 25%, both hydrolysates yielded relatively narrow peaks centered at *M*w of <3000 Da. For pepsin hydrolysates, quite different elution profiles were found. For DH of 5% hydrolysates, the content of the fraction with *M*w > 10 kDa was relatively low, indicating the low aggregation ratio comparing to alcalase and papain hydrolysates. For DH of 15% and 25% hydrolysates, higher contenst of fractions with *M*w ranges of 10,000–3000, 3000–1000 and 1000–500 Da were found. This was probably the result of the different selectivity of different proteolytic enzymes. Pepsin showed narrower selectivity than alcalase and papain.

**Figure 2:**
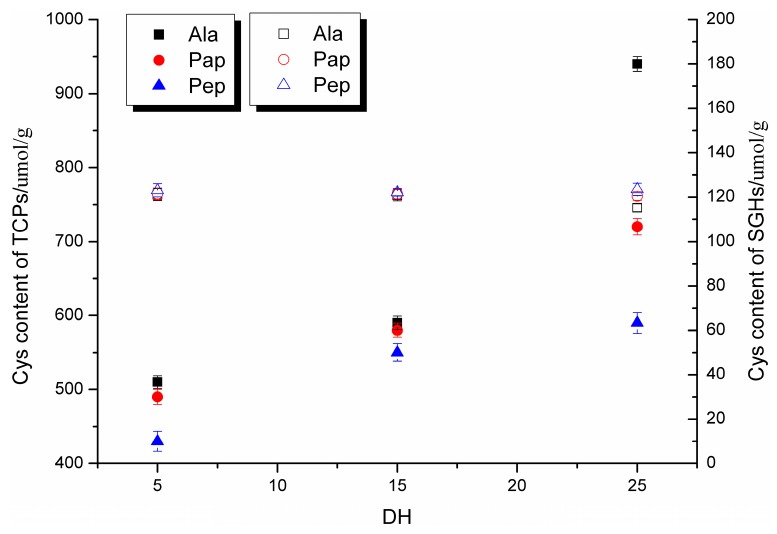
Total sulfhydryl group content of SGHs (□, ○, Δ) and sulfhydryl group content of thiol-containing peptides (TCPs) (■, ●, ▲) with different DH. Total sulfhydryl group content of SGHs was determined after reduction of disulfide bonds.

After reduction of the disulfide bonds of SGHs, the resultant TCPs were enriched by covalent chromatography on Thiopropyl-Sepharose 6B. As shown in Figure 2, the total sulfhydryl group contents in SGHs (μmol/g proteins) were essentially independent of enzymes and degree of hydrolysis, indicating that the loss of sulfhydryl group during proteolysis was slight. Sulfhydryl group contents after Thiopropyl-Sepharose 6B treatment increased substantially, as high as 8.2-fold observed in DH 25% alcalase hydrolysates. It is also remarkable to find that sulfhydryl group contents of TCPs were affected strongly by the enzyme and DH. For alcalase hydrolysates, sulfhydryl group enrichments were of 4.2-, 4.9-, and 8.2-fold for DH of 5%, 15% and 25%, respectively. While those of papain and pepsin hydrolysates were 4.0-, 4.5- and 4.8-fold and 3.5-, 4.5- and 4.8-fold, respectively. The different sulfhydryl group enrichments might be due to the different length of TCPs.

**Figure 3:**
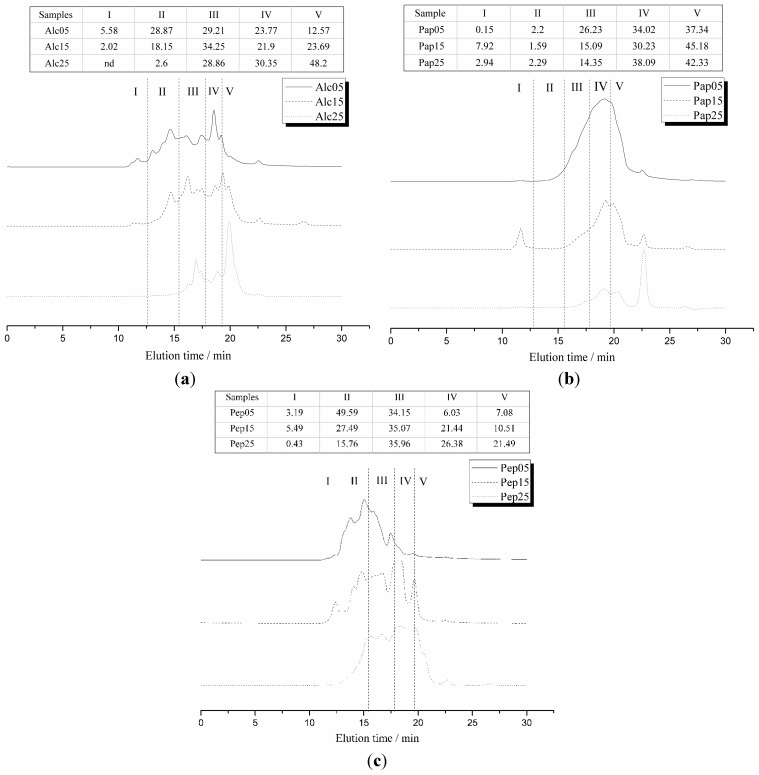
SEC-HPLC profiles of TCPs with different DH obtained by enzymatic hydrolysis with alcalase (**a**), papain (**b**) and pepsin (**c**).

The *M*w distributions of enriched TCPs as shown in Figure 3 were substantially different from those of the original hydrolysates. For alcalase and papain hydrolysates, Thiopropyl-Sepharose 6B treatment resulted in a shift of *M*w distribution to low range for DH 5% TCPs, while a shift to middle *M*w was noticed in DH 15% and 25% TCPs. TCPs enrichment of pepsin hydrolysates did not result in a remarkable difference in *M*w distribution, except in the fraction of *M*w > 10 kDa of DH 5%, in which it decreased substantially. Cys residue is expected to distribute equally in all *M*w fractions since alcalase, papain, and pepsin are all non-specific enzymes. The remarkable decrease in the fraction of *M*w > 10 kDa after enrichment with covalent chromatography, indicated that Thiopropyl-Sepharose 6B might preferentially bind TCPs having middle to low *M*w, while peptides with large *M*w might had very weak interactions with the gel due to steric hindrance. Paulech *et al.* [26] found a preference of Thiopropyl-Sepharose 6B for acidic residues and increased hydrophilicity in the regions immediately up- or downstream of the reactive Cys according to the analysis of amino acid sequence of the obtained thiol-containing peptides. However, Mi *et al.* [35] observed that some peptides with high hydrophilicity or hydrophobicity were lost during the procedure via the analysis of the lost thiol-containing peptides. Until recently, there has been no report found on the effects of molecular weight on Thiopropyl-Sepharose 6B-TCPs interactions.

### 2.2. pH Titration Curves in the Absence of Metal Ions

**Figure 4:**
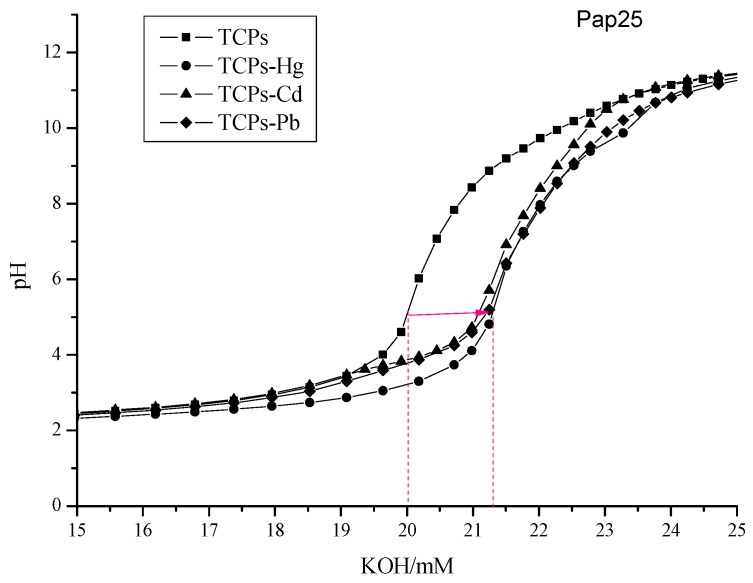
pH titration curves of TCPs and TCPs (■) in the presence of Hg^2+^ (●), Cd^2+^ (▲) or Pb^2+^ (◆) at 25 °C, I = 0.1 M KCl. The red solid line pointing right emphasizes the change of inflexions of TCPs after presence of heavy metals. The distance between the dotted lines gives the extent of the change.

In a classical pH titration experiment, pH changes are followed during the addition of increasing amounts of KOH to a solution containing 10 mM HCl and 4 mM TCPs, in the presence or absence of 1 mM Hg^2+^, Cd^2+^, or Pb^2+^. Figure 4 shows the pH titration curve of TCPs derived from DH 25% papain SGHs. The inflexion of the titration curve without metal ions is at about a total addition of 20 mM KOH. Since there was 10 mM HCl in the system, the other 10 mM proton could only be contributed to by carboxyl groups. The *C* terminal contributes at most 4 mM carboxyl for peptides containing 4 mM sulfhydryl groups, and the remaining 6 mM carboxyl can only be found on side chains of Asp and/or Glu residues. In the same way, carboxyl groups on TCPs from other hydrolysates were also obtained from the analysis of inflexions (Table 1). It was found that both DH 5% and DH 15% papain SGHs gave TCPs with about eight carboxyl groups, which was contradictory to the general assumption that longer peptides possess more acidic amino acids residues. A possible reason might be that more acidic thiol-containing peptides were recovered from DH 25% papain SGHs. The carboxyl groups calculated from inflexion points were about 4, 8, and 12 as well as 7, 8, and 12 from DH 25%, 15% and 5% for alcalase and pepsin hydrolysates, respectively. Therefore, for a fixed number of thiol groups, the carboxyl groups of alcalase and pepsin hydrolysates ran roughly parallel with the increase of peptide length.

**Table 1:** Inflections ^α^ of titration curves of TCPs in the absence of metal ions.

Hydrolysates	Alc05	Alc15	Alc25	Pap05	Pap15	Pap25	Pep05	Pep15	Pep25
Concentration of KOH (mM)	22.0	18	14	18.2	18.8	20.0	22.0	18.0	17.0

^α^ points having the largest inclined rate in the titration curves.

Using a nonlinear least square method for the pH titration curves, the dissociation constants (pK) were obtained as shown in Table 2 and Table 3. Due to the interactions among the groups, clear equivalence points could not be seen but it is possible to extract pK values from the titration curve. The pK values of glutathione were 2.3, 3.6, 8.9, and 9.8, which were about 0.2 units higher than those reported in the literature [36,37,38]. The number of titratable protons in the TCPs was markedly affected by both DH and type of enzyme. There were 6, 4, and 3 titratable protons in the TCPs obtained from alcalase hydrolysates as DH increased from 5% to 25%. While all TCPs from papain hydrolysates yielded four titratable protons irrespective of DH. For pepsin hydrolysates, titratable protons decreased from six to four when DH increased from 5% to 15%, but no change was observed on further increasing DH to 25%. It was also found that the numbers of titratable protons in the acidic range generally agreed with those obtained from the analysis of inflexion points. For any specific TCPs, the lowest pK, with values ranging from pH 1.8 to 3.6, corresponded to the *C*-terminal carboxylic acid. Khoury *et al.* [39], who studied the acid dissociation constants of tri-peptides containing Glu, Gly, and His using potentiometry, reported that pK values for *C*-terminal carboxylic acid ranged from pH 1.85 to 3.49. These authors also found that side chain pK values of Glu were also extensively affected by their position in the peptides, ranging from pH 3.73 to 6.81. In fact, it has long been known that the dissociation of the carboxyl group is greatly influenced by the electrostatic effect of the positive charges on amino and imidazole groups [40]. Titratable protons in the basic range were contributed to by the terminal amine amine and sulfhydryl groups of Lys and Arg residues. In a thorough study of cysteine ionization and reactivity, the pK values of Cys residues in 16 model peptides with both termini blocked were found to range from 7.4 to 9.1 [41]. Similarly, Khoury *et al.* [41] reported that the pK values of the terminal amine groups ranged from pH 7.23 to 9.54. Therefore, terminal amine and sulfhydryl groups are not distinguishable in their pK values. Zhang and Vogel [42] reported that the side chain pK values of the lysine residues in calmodulin ranged from 9.87 to 10.55. The pK of arginine’s side chain in a model peptide of Gly–Gly–Arg–Gly–Gly was determined to be 12.1. In this study, all the 12 TCPs gave titratable protons around pH 7–8 and some at pH higher than 10, suggesting that they contained both acidic and basic amino acid residues. A survey of the amino acid sequence of glycinin’s acidic polypeptides showed that in all 25 Cys residues, 22 of them are separated from Glu or Asp by less than five amino acid residues, while 20 of them are separated from Lys or Arg by less than five amino acid residues. Thus it is very likely to obtain TCPs possessing both acidic and basic amino acid residues.

**Table 2:** Experimentally determined glutathione acid dissociation constants and literature values at 25 °C, ionic strength (I) = 0.1 M KCl.

pK		pK_1_	pK_2_	pK_3_	pK_4_
Experimental Values		2.3	3.6	8.9	9.8
Literature Values	[35]	2.12	3.53	8.66	9.62
	[36]	2.09	3.48	8.67	9.54
	[37]	1.98	3.49	8.75	9.69

**Table 3:** Dissociation constants of TCPs at 25 °C, I = 0.1 M KCl.

DH/%	Alcalase	Papain	Pepsin
5	pK_1_ = 11.6, pK_2_ = 9.9, pK_3_ = 7.1, pK_4_ = 4.5, pK_5_ = 3.5, pK_6_ = 2.4	pK_1_ = 10.7, pK_2_ = 7.5, pK_3_ = 4.4, pK_4_ = 3.1	pK_1_ = 11.4, pK_2_ = 9.7, pK_3_ = 7.0, pK_4_ = 4.6, pK_5_ = 3.7, pK_6_ = 2.6
15	pK_1_ = 9.9, pK_2_ = 7.1, pK_3_ = 4.3, pK_4_ = 3.2	pK_1_ = 10.4, pK_2_ = 7.4, pK_3_ = 3.8, pK_4_ = 2.7	pK_1_ = 10.6, pK_2_ = 7.9, pK_3_ = 4.9, pK_4_ = 3.6
25	pK_1_ = 11.4, pK_2_ = 8.0, pK_3_ = 3.1	pK_1_ = 11.1, pK_2_ = 8.0, pK_3_ = 2.7, pK_4_ = 1.8	pK_1_ = 10.9, pK_2_ = 8.0, pK_3_ = 4.4, pK_4_ = 3.0

### 2.3. pH Titration Curves in the Presence of Metal Ions

In the presence of Hg^2+^ ions, the buffering capacity of TCPs from the DH 25% papain SGH in the alkaline region became smaller and the inflexion shifted to the right (Figure 4), confirming the formation of complexes between TCPs and metal ions. The metal titration curves are displaced to the right hand side of the ligand titration curves along the volume axis, indicating proton release upon complex formation of the metal ion with the ligand. Compared to the results for 4 mM TCPs alone, the maximum shift of the titration curve in the presence of 1 mM metal ions to the right was approximately 1.5 mM KOH. It could be deduced that some ligand molecules might be deprotonated twice by one metal ion or one metal ion might deprotonate two ligand molecules simultaneously. The decrease of pH for titration curves in the presence of metal ions also indicated the formation of metal complexes. The largest pH decrease was found to occur between pH 8.0 and 8.5, suggesting the important contribution of sulfhydryl groups in the complexation of metal ions with TCPs. Although TCPs contain a number of potentially coordinating sites, including amide nitrogen, carbonyl oxygen, *N*-terminal amino, and *C*-terminal carboxyl groups, the sulfhydryl groups are expected to be the primary binding groups to mercury (II) in the formation of a stable peptide-mercury (II) complex because of their preferential soft *S*-donor and soft Hg-acceptor interactions [15]. Chakraborty *et al.* [43] also suggested that Hg^2+^ preferred to undergo strong complexation reactions with reduced sulfur sites. The inflections of other titration curves of TCPs in the presence of metal ions shifted to the right, indicating that all TCPs prepared in this study bound metal ions. The average number of ligand molecules bound by each metal ion or $\bar{\text{n}}$, possessed values between zero and two, indicating the formation of ML and ML_2_ complexes in solution.

**Table 4:** Stability constants between TCPs and Hg^2+^, Cd^2+^ and Pb^2+^ at 25 °C, I = 0.1 M KCl ^α^.

Hydrolysates	Hg^2+^		Cd^2+^		Pb^2+^	
	lgβ_1_	lgβ_2_	lgβ_1_	lgβ_2_	lgβ_1_	lgβ_2_
Alc05	10.4 ± 0.5	15.4 ± 0.7	12.3 ± 0.6	18.7 ± 0.9	3.8 ± 0.2	7.8 ± 0.4
Alc15	16.0 ± 0.7	21.1 ± 1.0	11.4 ± 0.5	17.4 ± 0.7	10.1 ± 0.6	16.5 ± 0.8
Alc25	18.0 ± 0.3	30.5 ± 1.3	14.8 ± 0.7	25.3 ± 1.2	14.1 ± 0.7	25.6 ± 1.1
Pap05	17.5 ± 0.8	21.8 ± 0.9	7.8 ± 0.4	12.8 ± 0.6	7.6 ± 0.5	12.4 ± 0.8
Pap15	16.8 ± 0.8	21.2 ± 0.9	7.9 ± 0.4	12.3 ± 0.6	11.7 ± 0.5	17.2 ± 0.7
Pap25	19.4 ± 0.4	33.4 ± 1.5	9.2 ± 0.4	15.2 ± 0.8	13.3 ± 0.6	21.1 ± 0.9
Pep05	11.6 ± 0.6	16.0 ± 1.0	10.1 ± 0.5	14.6 ± 0.7	10.4 ± 0.5	15.6 ± 0.8
Pep15	24.4 ± 1.0	27.2 ± 0.9	11.7 ± 0.5	15.1 ± 0.8	10.5 ± 0.5	15.2 ± 0.7
Pep25	10.6 ± 0.3	33.1 ± 1.0	12.1 ± 0.6	17.1 ± 0.8	12.6 ± 0.6	18.0 ± 0.7

^α^ Values expressed as means of triplicate ± standard error.

Experimental values of formation constants for Hg^2+^-GSH complex in this study were 10^19.2^ and 10^30.5^. These results were in line with those presented by Oram *et al.* [44]. However, very different Hg^2+^-GSH formation stability constants were reported by Cardiano *et al.* [31]. Leverrier *et al.* [32] reported that stability constants of CdGSH and Cd(GSH)_2_ were 10^8.5^ and 10^12.4^, respectively, which did not deviate far from our results (10^10.1^ and 10^15.4^, respectively). The complexing stability constants, which were read from the formation curves, are shown in Table 4. TCPs exhibited high binding affinities for heavy metal ions. In addition, the binding affinities increased as the DH increased from 5% to 25%. TCPs from DH 25% hydrolysates gave similar or even larger lgβ values compare to glutathione. The order of the stability of complexes between TCPs and heavy metals was found to be in the order of Hg^2+^ > Cd^2+^ > Pb^2+^ for alcalase and pepsin TCPs and Hg^2+^ > Pb^2+^ > Cd^2+^ for papain TCPs with the same tendency as those of GSH. The stability constant value varies with the ionic size of the metal ion. The smaller the ionic radius of the central atom, the more stable is the complex formed. However, the ionic radius in this experiment was in the order of Cd^2+^ < Hg^2+^ < Pb^2+^. It may be dependent upon the experimental conditions used. The similar metallic ions complexing behavior could be partly explained by the fact that TCPs had similar proton dissociation constants to those of GSH’s. The results indicated that TCPs might be promising alternatives for GSH as chelating agents for heavy metal ions.

### 2.4. Correlations between Sulfhydryl Group Content and Stability Constants

**Figure 5:**
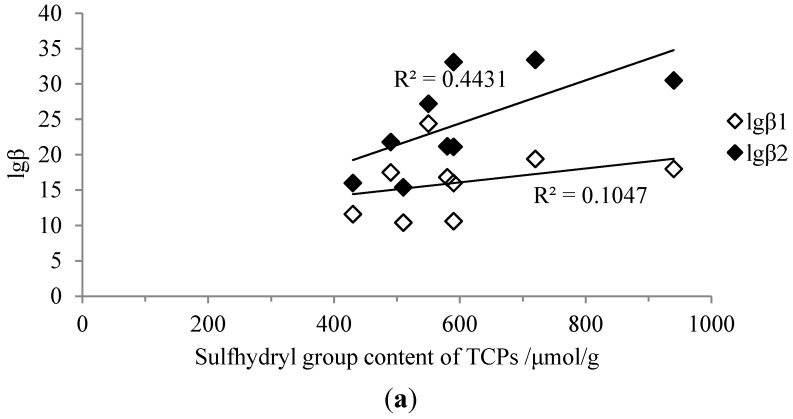

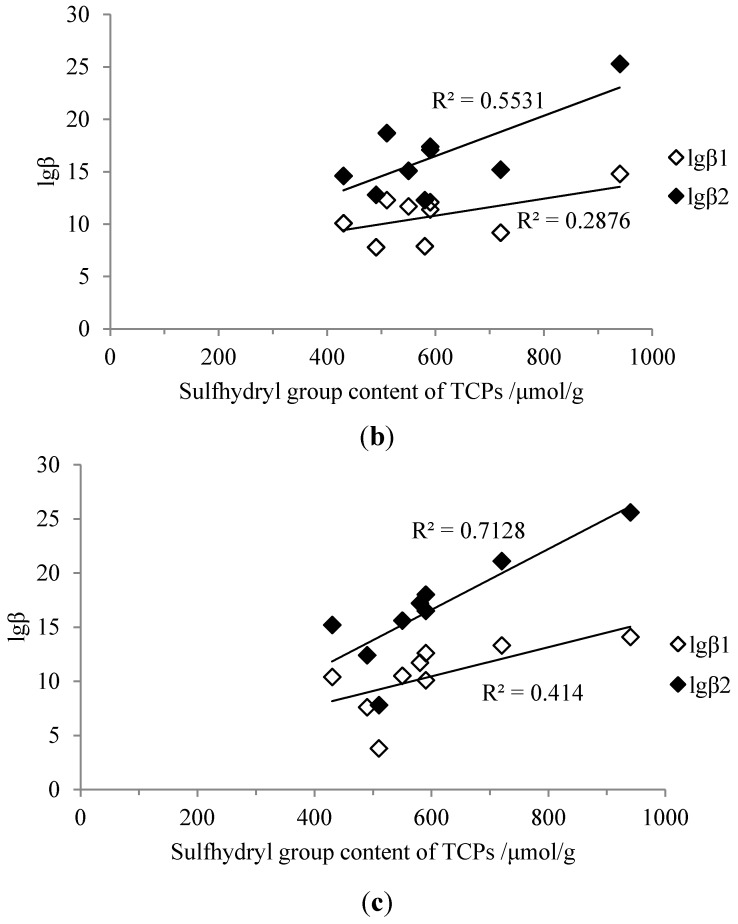
Correlations between content of sulfhydryl group and stability constants. (**a**) Hg^2+^; (**b**) Cd^2+^; (**c**) Pb^2+^.

The correlations between sulfhydryl group content and the logarithm of stability constants (lgβ) of TCPs-metallic ion complexes are shown in Figure 5. The correlation coefficients (*r*) between sulfhydryl group contents and lgβ_1_ were 0.324, 0.536, and 0.643 for Hg^2+^, Cd^2+^, and Pb^2+^ complexes, respectively. This indicated that there was a moderate linear dependence between sulfhydryl group content and lgβ_1_. Significant positive correlations existed between sulfhydryl group contents and the lgβ_2_, with r being 0.666 (*p* < 0.05), 0.744 (*p* < 0.05) and 0.844 (*p* < 0.01) for Hg^2+^, Cd^2+^ and Pb^2+^ complexes, respectively. The results suggested that the heavy metal chelating ability of TCPs was not only affected by the content of the sulfhydryl group, but also depended on the global structure of the peptides. The significant correlation between lgβ_2_ and sulfhydryl group content indicated that sulfhydryl groups played more important roles in forming ML_2_ than other groups, possibly because the Cys *S*-donor can readily form a linear, two S coordination complex with mercury (II) (ML_2_) [32]. On the other hand, other groups, such as the imidazole of the histidyl residue could form tetrahedral 2 *N*- and 2 *S*-coordinated ML_2_ complexes, which are relatively less stable due to steric hindrance [32].

### 2.5. Correlations between Mw Distributions and Stability Constants

Table 5 shows the correlations between *M*w distributions and lgβ. In all cases, lgβ is inversely related to the contents of fraction I and II and directly related to the contents of IV and V. The lgβ showed poor linear relationship with fraction III. For Hg^2+^, lgβ_2_ significantly related (*r* = 0.815, *p* < 0.01) to fraction IV. For Cd^2+^, no significant linear relationship existed between any fraction and lgβ. For Pb^2+^, both lgβ_1_ and lgβ_2_ significantly related to fraction I (*r* = −0.854, *p* < 0.01 for lgβ_1_ and *r* = −0.762, *p* < 0.05 for lgβ_2_), IV (*r* = 0.702, *p* < 0.05 for lgβ_1_ and *r* = 0.689, *p* < 0.05 for lgβ_2_) and V (*r* = 681, *p* < 0.05 for lgβ_1_ and *r* = 0.835, *p* < 0.01 for lgβ_2_). These results indicated that the *M*w distribution affected interactions between TCPs and Pb^2+^ more notably than the other two metal ions. The influence of *M*w distribution on interactions between TCPs and heavy metal ions may be due to the steric hindrance between TCPs and metal ions.

**Table 5:** Correlation coefficients between *M*w distribution and stability constants.

Heavy Metals	Fraction I		Fraction II		Fraction III		Fraction IV		Fraction V	
	lgβ_1_	lgβ_2_	lgβ_1_	lgβ_2_	lgβ_1_	lgβ_2_	lgβ_1_	lgβ_2_	lgβ_1_	lgβ_2_
Hg^2+^	−0.2	−0.581	−0.202	−0.395	−0.097	0.122	0.410	0.815 ^b^	0.279	0.553
Cd^2+^	−0.379	−0.274	−0.028	−0.273	0.187	−0.154	0.197	0.229	0.243	0.511
Pb^2+^	−0.854 ^b^	−0.762 ^a^	−0.142	−0.300	0.159	−0.057	0.702 ^a^	0.689 ^a^	0.681 ^a^	0.835 ^b^

^a^ Correlation is significant at the 0.05 level; ^b^ Correlation is significant at the 0.01 level.

## 3. Experimental Section

### 3.1. Materials

Low-denatured, defatted soy flakes were kindly provided by Shandong Gushen Industrial & Commercial Co., Ltd. (Dezhou, China). The flakes had a protein content of 53.13% (*N* × 6.25, dry base) and nitrogen solubility index of 87%. β-mercaptoethanol (2-ME) was purchased from Shanghai Shengzheng Biotech. Co., Ltd. (Shanghai, China). Alcalase 2.4 L FG (180,000 U/mL) was purchased from Novozymes (Beijing, China) Biological Technology Co. Papain, Pepsin and 4,4'-dithiodipyridine (4-DPS) were purchased from Sigma-Aldrich Trading Co., Ltd. (Shanghai, China). DTT and C 18 resin were from Merck (Beijing, China). Sephadex LH-20 and Thiopropyl-Sepharose 6B resins were purchased from GE Healthcare Life Sciences (Shanghai, China). Chromatographic grade acetonitrile was purchased from J&K China Chemical Ltd. (Shanghai, China). All other chemicals were of analytical grade.

### 3.2. Preparation of Soy Glycinin

Soy glycinin (11S) was isolated from defatted soybean flakes according to the method of Wolf [23] without purification. The defatted soybean flakes were suspended in a 30 mM Tris-HCl buffer, pH 8.0, containing 10 mM 2-mercaptoethanol with a flake to buffer ratio of 1:10 (*w/v*) and stirred at ambient temperature for 1 h. After removal of the insoluble parts by centrifugation (30 min, 12,000× *g*, 4 °C), the supernatant was brought to pH 6.4 with 2 M HCl to induce precipitation of the proteins and centrifuged for 20 min (12,000× *g*, 4 °C). The protein precipitate was re-suspended in water and adjusted to pH 8.0 with 2 M NaOH. The protein dispersion was dialysed thoroughly against distilled water to remove the salt and 2-mercaptoethanol. The pH of the dialyzed protein dispersion was adjusted to pH 8.0 with 2 M NaOH and the clear solution obtained was freeze-dried and stored at 4 °C. Protein content of the prepared 11S was (95.3 ± 1.6)% (*w/w*) as determined by the micro-Kjeldahl method with a nitrogen conversion factor of 6.25. The purity of the 11S was (90 ± 3.1)% according to SDS-PAGE.

### 3.3. Enzymatic Hydrolysis of 11S

Hydrolysis of the 11S using alcalase, papain, and pepsin was conducted under the following conditions: 50 °C, pH 8.0; 50 °C, pH 7.0, and 37 °C, pH 2.0. Prior to each hydrolysis experiment, protein solution was freshly prepared by suspending the freeze-dried 11S in deionized water to a concentration of 10.0% (*w/w*). The dispersion was stirred overnight at 4 °C. After equilibration to ambient temperature the pH, it was readjusted to 8.0. The solution was centrifuged (30 min, 22,000× *g*, 4 °C) to remove any insoluble proteins and the supernatant was diluted to a concentration of 5% (*w/w*). After adjusting the temperature and pH to the desired values, proteolytic enzyme was added at an enzyme to substrate ratio (E/S) of 5:100 to initiate hydrolysis. Digestions were performed according to the pH-stat method using 0.5 M standard NaOH for alcalase and papain and 1 M standard HCl for pepsin. Hydrolysis was stopped at the degree of hydrolysis of 5%, 15% and 25% respectively by heating the reactant in boiling water for 5 min to inactivate the enzymes. The suspension was centrifuged at 10,000× *g* for 15 min and the supernatant was lyophilized and stored at 4 °C until use.

### 3.4. Sulfhydryl Group Content Measurement

Sulphydryl group content was determined using 4,4'-dithiodipyridine (4-DPS) according to Riener *et al*. [45] with minor modification. Aliquots (0.3 mL) of TCPs solutions (about 25 μmol/L) were mixed with 2.7 mL 0.1 M citrate-Na_2_HPO_4_ buffer (pH 4.5) containing 1% SDS and 125 μL 4-DPS (4 mM) solution. After 30 min standing at ambient temperature, the absorption of the mixture at 324 nm was determined. Soluble protein concentration was evaluated by the biuret method with BSA as the standard. The micromole sulfhydryl group per gram peptides was calculated by using the extinction coefficient of 21,400 M^−1^·cm^−1^. Sulfhydryl group content was expressed as μmol/g protein.

### 3.5. Total Sulfhydryl Group Measurement

Content of total sulfhydryl group was determined according to Cavallini *et al.* [46] with some modifications. The hydrolysate solution used for determination of sulfhydryl content was diluted to about 10 mg/mL. To 1 mL sample solution was added a 0.5 mL sodium borohydride (2.5%, dissolved in 1 M NaOH solution) and reduction was allowed to take place for 30 min at 50 °C. Excess sodium borohydride was removed by adding 0.15 M HCl. After this, the subsequent procedure was the same as in Section 2.4.

### 3.6. TCPs Extraction

TCPs enrichment was performed by combination of disulfide bond reduction in SGHs according to the method of Wolf [23] and batch-based covalent chromatography using Thiopropyl-Sepharose 6B [29]. Briefly, hydrolysates solution at a concentration of 50 g/L was reacted with 30 mM DTT at pH 8.0 and 50 °C for 30 min. The solution was then adjusted to pH 3.0 and subsequently applied to the Sephadex LH-20 column, which was equilibrated and eluted with 1.0 mM HCl to remove the excess DTT. Thiopropyl-Sepharose 6B beads were rehydrated in Milli-Q water and washed by 50 bed volumes of water. Samples eluted from Sephadex LH-20, after adjusting to pH 7.5 and adding 0.5% SDS, were applied to the beads immediately and allowed to bind at room temperature for 2 h with gentle tumbling in the protection of purified nitrogen. The resin and captured peptides were washed by 2 bed volumes of 0.5% SDS and 10 bed volumes of water to remove the unbound fraction. The captured peptides were liberated with 25 mM DTT in 10 mM Tris-HCl, pH 7.5. Excess DTT and 2-thiopyridine in the elution was removed by C-18 column.

### 3.7. Molecular Weight Distribution by Size Exclusion Chromatography

The molecular weight distribution of SGHs and TCPs was analyzed on an Elite L-2000 HPLC (Hitachi, Tokyo, Japan) using a TSK gel 2000 SW_XL_ column (300 × 7.8 mm i.d., Tosoh, Tokyo, Japan) [47]. The column was eluted with acetonitrile/water/trifluoroacetic acid = 45/55/0.1 (*v*/*v*) at a flow rate of 0.5 mL/min and the eluent was monitored at 214 nm. The calibration curve was prepared from the average retention times of the molecular weight standard containing cytochrome C (12,500 Da), bacitracin (1450 Da), tetrapeptide GGYR (451 Da) and tripeptide GGG (189 Da).

### 3.8. Potentiometric Determinations

The potentiometric titration cell was assembled in the laboratory consisting of a stirred, thermostatically controlled 50 mL reactor covered with a Teflon-wrapped rubber stopper, a high-precision pH probe connected to a pH meter (Model FE 20; Mettler-Toledo Instruments Co., Ltd., Shanghai, China) and an inlet and an outlet of nitrogen gas. The pH meter was calibrated with standard buffers, *i.e.*, pH 4.00 (0.05 M potassium biphthalate), pH 6.86 (0.025 M mixed phosphate buffer) and pH 9.18 (0.01 M sodium tetraborate decahydrate). Titrations were performed at 25 ± 0.1 °C over the pH range of 2.0–12.0, with 0.1 M KOH as the titrant. For each titration, sample volume was maintained at 25 mL with TCP and heavy metal ion concentrations being fixed at 4 and 1 mM. The concentration of TCPs was the same as that of sulfhydryl groups on the assumption that one TCP molecule has one sulfhydryl group. Titration curves were obtained by plotting concentration of KOH added against pH values.

### 3.9. Calculation of Stability Constants

The calculation of stability constants of TCP-heavy metal complexes was conducted according to the method of Bjerrum [48]. The overall stability constants for M + nL = ML_n_ are designated by the expression of β_n_ = [ML_n_]/[M][L]^n^, and those for L + jH = LH_j_ are β_j_^H^ = [LH_j_]/[L][H]^j^, where β_n_, β_j_^H^, L, M and H stand for metal-ligand complex stability constants, protonation constans, ligand, metal ion, and proton, respectively. All charges on ligands, metal ions, and the complexes are omitted for the sake of simplicity.

Let the total concentration of ligands, metal ions, and ionisable hydrogen in the system be C_L_, C_M_, and C_H_, respectively. Then, at any point of equilibrium, average number of protons bound by each ligand is:

(1) $${\bar{\text{n}}}_{\text{H}}=\frac{\text{total concentration of proton bound to ligand}}{\text{total concentration of ligand not bound to the metal }}=\frac{{\text{jC}}_{\text{L}}+{\text{C}}_{\text{A}}+\left[\text{OH}\right]-\left[\text{Na}\right]-[\text{H}]}{\left[\text{L}\right]+\left[\text{HL}\right]+\left[{\text{H}}_{2}\text{L}\right]+\ldots }$$

for ligand solutions in the absence of metal ions,

(2) $$\left[\text{L}\right]+\left[\text{HL}\right]+\left[{\text{H}}_{2}\text{L}\right]+\ldots ={\text{C}}_{\text{L}}$$

(3) $${\bar{\text{n}}}_{\text{H}}=\frac{\text{total concentration of proton bound to ligand}}{\text{concentration of ligand not bound to the metal}}=\frac{\left[\text{HL}\right]+2\left[{\text{H}}_{2}\text{L}\right]+\ldots }{\left[\text{L}\right]+\left[\text{HL}\right]+\left[{\text{H}}_{2}\text{L}\right]+\ldots }=\frac{{\text{β}}_{1}^{\text{H}}\left[\text{H}\right]+2{\text{β}}_{2}^{\text{H}}{\left[\text{H}\right]}^{2}+\ldots +{\text{jβ}}_{\text{j}}^{\text{H}}{\left[\text{H}\right]}^{\text{j}}}{1+{\text{β}}_{1}^{\text{H}}\left[\text{H}\right]+{\text{β}}_{2}^{\text{H}}{\left[\text{H}\right]}^{2}+{\text{β}}_{\text{j}}^{\text{H}}{\left[\text{H}\right]}^{\text{j}}}$$

Protonation constans (β_j_^H^) were calculated from formula 1, 2, and 3 according to the titration data of ligand solutions in the absence of metal ions by using the nonlinear least square method. The corresponding proton dissociation constant Kj is the reciprocal of β_j_^H^, while pKj = −log Kj.

Similarly, average number of ligand molecules bound by each metal ion is:

(4) $$\bar{\text{n}}=\frac{\text{total concentration of ligand bound to metal}}{\text{total concentration of metal}}=\frac{{\text{C}}_{\text{L}}-(\left[\text{L}\right]+[\text{HL}]+\ldots [{\text{H}}_{\text{j}}\text{L}]}{{\text{C}}_{\text{M}}}$$

or

(5) $$\bar{\text{n}}+\left(\bar{\text{n}}-1\right){\text{β}}_{1}\left[\text{L}\right]+\left(\bar{\text{n}}-2\right){\text{β}}_{2}{\left[\text{L}\right]}^{2}+\ldots \left(\bar{\text{n}}-\text{n}\right){\text{β}}_{\text{n}}{\left[\text{L}\right]}^{\text{n}}=0$$

From formula1, the following was obtained:

(6) $$[\text{L}]+[\text{HL}]+\ldots [\text{HjL}]=\frac{{\text{jC}}_{\text{L}}+{\text{C}}_{\text{A}}+\left[\text{OH}\right]-\left[\text{Na}\right]-[\text{H}]}{{\bar{\text{n}}}_{\text{H}}}$$

Then

(7) $$[\text{L}]=\frac{{\text{jC}}_{\text{L}}+{\text{C}}_{\text{A}}+\left[\text{OH}\right]-\left[\text{Na}\right]-[\text{H}]}{{\text{β}}_{1}^{\text{H}}\left[\text{H}\right]+2{\text{β}}_{2}^{\text{H}}{\left[\text{H}\right]}^{2}+\ldots +{\text{jβ}}_{\text{j}}^{\text{H}}{\left[\text{H}\right]}^{\text{j}}}$$

Combining 3 and 5 the following was obtained:

(8) $$\bar{\text{n}}=\frac{{\text{C}}_{\text{L}}-\frac{{\text{jC}}_{\text{L}}+{\text{C}}_{\text{A}}+\left[\text{OH}\right]-\left[\text{Na}\right]-[\text{H}]}{{\bar{\text{n}}}_{\text{H}}}}{{\text{C}}_{\text{M}}}$$

The half integral method was applied to obtain the metal-ligand complex stability constants. By plotting $\bar{\text{n}}$ against pL (−log[L]), a complex ligand formation curve was obtained. The stepwise stability constants (K*n*) were calculated from the formation curve by the known values of pL at which $\bar{\text{n}}$ = 1 – 1/2, 2 – 1/2··· corresponds to the values of logK_n_. The overall metal-ligand complex stability constants were calculated as β_n_ = K_1_·K_2_···K_n_.

### 3.10. Statistical Analysis

Experiment data were analyzed by analysis of variance (ANOVA) using the SPSS package. A least significant difference (LSD) test with a confidence interval of 95% was used to compare the means. All treatments were run in triplicate. Data were expressed as the mean ± SD (*n* = 3). Pearson’s correlation coefficient was used to express the correlation.

## 4. Conclusions

In conclusion, TCPs extracted from SGHs possessed high sequestering abilities for Hg^2+^, Cd^2+^, and Pb^2+^. TCPs from DH 25% SGHs were as effective as glutathione. Their binding affinities for heavy metal were not only affected by the content of sulfhydryl group but also the molecular weight distribution. The sulfhydryl group content related positively to lgβ_2_ of the TCPs-Hg^2+^ complex. The content of fractions with *M*w > 10,000 Da related inversely, while the content of fractions with *M*w < 1000 Da related positively to lgβ_1_ and lgβ_2_ of the TCPs-Hg^2+^ complex. Therefore, soy TCPs could be used as ingredients in the formulation of functional foods to control and manage diseases associated with heavy metal accumulation. TCPs here were a mixture of various peptides, thus stability constants given in this work were approximate. Further purification needs to be carried out to gain exact stability constants.
